# General fractional integral inequalities for convex and *m*-convex functions via an extended generalized Mittag-Leffler function

**DOI:** 10.1186/s13660-018-1830-8

**Published:** 2018-09-15

**Authors:** G. Farid, K. A. Khan, N. Latif, A. U. Rehman, S. Mehmood

**Affiliations:** 1 Department of Mathematics, COMSATS University Islamabad, Attock, Pakistan; 2 Department of Mathematics, University of Sargodha, Sargodha, Pakistan; 3 General Studies Department, Jubail Industrial College, Jubail, Kingdom of Saudi Arabia; 4 GBPS Sherani, Hazro Attock, Pakistan

**Keywords:** 26A51, 26A33, 33E12, Convex function, *m*-convex function, Mittag-Leffler function, Generalized fractional integral operators, Hadamard inequality

## Abstract

In this paper some new general fractional integral inequalities for convex and *m*-convex functions by involving an extended Mittag-Leffler function are presented. These results produce inequalities for several kinds of fractional integral operators. Some interesting special cases of our main results are also pointed out.

## Introduction, definitions, and preliminaries

Convex functions are very important in the field of integral inequalities. A lot of fractional integral inequalities and novel results have been established due to convex functions (for more details, see [1, 8, 13, 14]).

### Definition 1

A function $f: I\rightarrow\mathbb{R}$, where *I* is an interval in $\mathbb{R}$, is said to be a convex function if

1 $$ f\bigl(tx+(1-t)y\bigr)\leq tf(x)+(1-t)f(y) $$

holds for $t\in[0,1]$ and $x,y\in I$.

A convex function $f: I\rightarrow\mathbb{R}$ is also equivalently defined by the Hadamard inequality

$$ f \biggl(\frac{a+b}{2} \biggr)\leq\frac{1}{b-a} \int_{a}^{b}f(x)\,dx \leq\frac{f(a)+f(b)}{2}, $$

where $a,b\in I$, $a< b$.

The concept of *m*-convexity was introduced in [17] and since then many properties, especially inequalities, have been obtained for this class of functions (see [3, 6, 7, 18]).

### Definition 2

A function $f:[0,b]\rightarrow\mathbb{R}$, $b>0$ is called *m*-convex, where $m\in[0,1]$, if for every $x,y\in[0,b]$ and $t\in[0,1]$, we have

$$ f \bigl(tx+m(1-t)y \bigr)\leq tf(x)+m(1-t)f(y). $$

For $m=1$, we recapture the definition of convex functions, and for $m=0$, the definition of star-shaped functions defined on $[0,b]$. We recall that a function $f:[0,b]\to\mathbb{R}$ is called *star-shaped* if

$$f(tx)\leq tf(x)\quad \mbox{for all } t\in[0,1] \mbox{ and } x\in[0,b]. $$

If we denote by $K_{m}(b)$ the set of *m*-convex functions defined on $[0,b]$ for which $f(0)<0$, then

$$K_{1}(b) \subset K_{m}(b) \subset K_{0}(b), $$

whenever $m\in(0,1)$. Note that in the class $K_{1}(b)$ there are only convex functions $f:[0,b]\to\mathbb{R}$ for which $f(0)\leq0$ (see [4]), while $k_{0}(b)$ contains *star-shaped* functions.

### Example 1.1

([6])

The function $f:[0,\infty)\to\mathbb{R}$, given by

$$f(x)=\frac{1}{12} \bigl(x^{4}-5x^{3}+9x^{2}-5x \bigr), $$

is a $\frac{16}{17}$-convex function but it is not *m*-convex for any $m\in(\frac{16}{17},1]$.

For more results and inequalities related to *m*-convex functions, one can consult, for example, [3, 6, 7] along with the references therein.

Recently in [2] Andrić et al. defined an extended generalized Mittag-Leffler function $E_{\mu,\alpha,l}^{\gamma,\delta,k,c}(\cdot;p)$ as follows.

### Definition 3

([2])

Let $\mu,\alpha,l,\gamma,c\in\mathbb{C}$, $\Re(\mu),\Re(\alpha ),\Re(l)>0$, $\Re(c)>\Re(\gamma)>0$ with $p\geq0$, $\delta>0$, and $0< k\leq\delta+\Re(\mu)$. Then the extended generalized Mittag-Leffler function $E_{\mu,\alpha,l}^{\gamma,\delta,k,c}(t;p)$ is defined by

2 $$ E_{\mu,\alpha,l}^{\gamma,\delta,k,c}(t;p)= \sum _{n=0}^{\infty }\frac{\beta_{p}(\gamma+nk,c-\gamma)}{\beta(\gamma,c-\gamma)} \frac{(c)_{nk}}{\Gamma(\mu n +\alpha)} \frac{t^{n}}{(l)_{n \delta}}, $$

where $\beta_{p}$ is the generalized beta function defined by

$$ \beta_{p}(x,y) = \int_{0}^{1}t^{x-1}(1-t)^{y-1}e^{-\frac {p}{t(1-t)}}\,dt $$

and $(c)_{nk}$ is the Pochhammer symbol defined as $(c)_{nk}=\frac {\Gamma(c+nk)}{\Gamma(c)}$.

In [2] properties of the generalized Mittag-Leffler function are discussed, and it is given that $E_{\mu,\alpha,l}^{\gamma,\delta,k,c}(t;p)$ is absolutely convergent for $k<\delta+\Re(\mu)$. Let *S* be the sum of series of absolute terms of the Mittag-Leffler function $E_{\mu,\alpha,l}^{\gamma,\delta,k,c}(t;p)$, then we have $\vert E_{\mu,\alpha,l}^{\gamma,\delta,k,c}(t;p) \vert \leq S$. We use this property of Mittag-Leffler function in our results where we need.

The corresponding left and right sided extended generalized fractional integral operators are defined as follows.

### Definition 4

([2])

Let $\omega,\mu,\alpha,l,\gamma,c\in\mathbb{C}$, $\Re(\mu),\Re(\alpha),\Re(l)>0$, $\Re(c)>\Re(\gamma)>0$ with $p\geq0$, $\delta>0$ and $0< k\leq \delta+\Re(\mu)$. Let $f\in L_{1}[a,b]$ and $x\in[a,b]$. Then the extended generalized fractional integral operators $\epsilon_{\mu,\alpha,l,\omega,a^{+}}^{\gamma,\delta,k,c}f $ and $\epsilon_{\mu,\alpha,l,\omega,b^{-}}^{\gamma,\delta,k,c}f$ are defined by

3 $$ \bigl( \epsilon_{\mu,\alpha,l,\omega,a^{+}}^{\gamma,\delta,k,c}f \bigr) (x;p)= \int_{a}^{x}(x-t)^{\alpha-1}E_{\mu,\alpha,l}^{\gamma,\delta,k,c} \bigl(\omega(x-t)^{\mu};p\bigr)f(t)\,dt $$

and

4 $$ \bigl( \epsilon_{\mu,\alpha,l,\omega,b^{-}}^{\gamma,\delta,k,c}f \bigr) (x;p)= \int_{x}^{b}(t-x)^{\alpha-1}E_{\mu,\alpha,l}^{\gamma,\delta,k,c} \bigl(\omega(t-x)^{\mu};p\bigr)f(t)\,dt. $$

From extended generalized fractional integral operators, we have

$$\begin{aligned} & (\epsilon_{\mu,\alpha,l,\omega,a^{+}}^{\gamma,\delta,k,c}1 )(x;p) \\ &\quad = \int_{a}^{x}(x-t)^{\alpha-1}E_{\mu,\alpha,l}^{\gamma,\delta,k,c}(w(x-t)^{\mu};p)\,dt \\ &\quad = \int_{a}^{x}(x-t)^{\alpha-1}\sum_{n=0}^{\infty}\frac{\mathrm{B}_{p}(\gamma+nk,c-\gamma)}{\mathrm{B}(\gamma,c-\gamma)}\frac {(c)_{nk}}{\Gamma(\mu n+\alpha)}\frac{w^{n}(x-t)^{\mu n}}{(l)_{n\delta}}\,dt \\ &\quad =\sum_{n=0}^{\infty}\frac{\mathrm{B}_{p}(\gamma+nk,c-\gamma )}{\mathrm{B}(\gamma,c-\gamma)}\frac{(c)_{nk}}{\Gamma(\mu n+\alpha)}\frac{w^{n} }{(l)_{n\delta}} \int_{a}^{x}(x-t)^{\mu n+\alpha-1}\,dt \\ &\quad = (x-a )^{\alpha}\sum_{n=0}^{\infty}\frac{\mathrm{B}_{p}(\gamma+nk,c-\gamma)}{\mathrm{B}(\gamma,c-\gamma)}\frac {(c)_{nk}}{\Gamma(\mu n+\alpha)}\frac{w^{n} }{(l)_{n\delta}} (x-a )^{\mu n}\frac{1}{\mu n + \alpha}. \end{aligned}$$

Hence

$$ \bigl(\epsilon_{\mu,\alpha,l,\omega,a^{+}}^{\gamma,\delta,k,c}1 \bigr) (x;p)= (x-a )^{\alpha} E_{\mu,\alpha +1,l}^{\gamma,\delta,k,c}\bigl(w(x-a)^{\mu};p \bigr), $$

and similarly

$$ \bigl(\epsilon_{\mu,\alpha,l,\omega,b^{-}}^{\gamma,\delta,k,c}1 \bigr) (x;p)= (b-x )^{\alpha} E_{\mu,\alpha +1,l}^{\gamma,\delta,k,c}\bigl(w(b-x)^{\mu};p \bigr). $$

We use the following notations in our results:

5 $$ C_{\alpha,a^{+}}(x;p)= \bigl(\epsilon_{\mu,\alpha,l,\omega,a^{+}}^{\gamma,\delta,k,c}1 \bigr) (x;p) $$

and

6 $$ C_{\alpha,b^{-}}(x;p)= \bigl(\epsilon_{\mu,\alpha,l,\omega,b^{-}}^{\gamma,\delta,k,c}1 \bigr) (x;p). $$

For more information related to Mittag-Leffler functions and corresponding fractional integral operators, the readers are referred to [9–12, 15, 16, 19].

In this paper we give general fractional integral inequalities for convex and *m*-convex functions by involving an extended Mittag-Leffler function and deduce some results already published in [1, 5, 6, 8, 13]. Also we give a Hadamard type inequality for convex and *m*-convex functions by involving an extended Mittag-Leffler function.

## Main results

Here we give some fractional integral inequalities for convex and *m*-convex functions via an extended generalized Mittag-Leffler function and corresponding fractional integral operators given in (3) and (4). The following lemma is useful to establish the results.

### Lemma 2.1

*Let* $f:[a,mb]\rightarrow\mathbb{R}$ *be a differentiable function such that* $f'\in L_{1}[a,mb]$ *with* $0\leq a< mb$. *Also let* $g:[a,mb]\rightarrow\mathbb{R}$ *be a continuous function on* $[a,mb]$, *then the following identity for extended generalized fractional integral operators holds*:

7 $$\begin{aligned} & \biggl( \int_{a}^{mb}g(s)E_{\mu,\alpha,l}^{\gamma,\delta,k, c} \bigl(\omega s^{\mu}; p\bigr)\,ds \biggr)^{\alpha}\bigl[f(a)+f(mb) \bigr] \\ &\qquad{} -\alpha \int_{a}^{mb} \biggl( \int_{a}^{t}g(s)E_{\mu,\alpha,l}^{\gamma,\delta,k, c} \bigl(\omega s^{\mu}; p\bigr)\,ds \biggr)^{\alpha-1} g(t)E_{\mu,\alpha,l}^{\gamma,\delta,k, c}\bigl(\omega t^{\mu}; p\bigr)f(t)\,dt \\ &\qquad{} -\alpha \int_{a}^{mb} \biggl( \int_{t}^{mb}g(s)E_{\mu,\alpha,l}^{\gamma,\delta,k, c} \bigl(\omega s^{\mu}; p\bigr)\,ds \biggr)^{\alpha-1} g(t)E_{\mu,\alpha,l}^{\gamma,\delta,k, c}\bigl(\omega t^{\mu}; p\bigr)f(t)\,dt \\ &\quad = \int_{a}^{mb} \biggl( \int_{a}^{t}g(s)E_{\mu,\alpha,l}^{\gamma,\delta,k, c} \bigl(\omega s^{\mu}; p\bigr)\,ds \biggr)^{\alpha}f'(t)\,dt \\ &\qquad{} - \int_{a}^{mb} \biggl( \int_{t}^{mb}g(s)E_{\mu,\alpha,l}^{\gamma,\delta,k, c} \bigl(\omega s^{\mu}; p\bigr)\,ds \biggr)^{\alpha }f'(t)\,dt. \end{aligned}$$

### Proof

On integrating by parts one can have

8 $$\begin{aligned} & \int_{a}^{mb} \biggl( \int_{a}^{t}g(s)E_{\mu,\alpha,l}^{\gamma,\delta,k,c} \bigl(\omega s^{\mu};p\bigr)\,ds \biggr)^{\alpha}f'(t)\,dt \\ &\quad = \biggl( \int_{a}^{mb}g(s)E_{\mu,\alpha,l}^{\gamma,\delta,k,c} \bigl(\omega s^{\mu};p\bigr)\,ds \biggr)^{\alpha}f(mb) \\ &\qquad {}-\alpha \int _{a}^{mb} \biggl( \int_{a}^{t}g(s)E_{\mu,\alpha,l}^{\gamma,\delta,k,c} \bigl(\omega s^{\mu};p\bigr)\,ds \biggr)^{\alpha-1} g(t)E_{\mu,\alpha,l}^{\gamma,\delta,k,c}\bigl(\omega t^{\mu };p \bigr)f(t)\,dt \end{aligned}$$

and

9 $$\begin{aligned} & \int_{a}^{mb} \biggl( \int_{t}^{mb}g(s)E_{\mu,\alpha,l}^{\gamma,\delta,k,c} \bigl(\omega s^{\mu};p\bigr)\,ds \biggr)^{\alpha}f'(t)\,dt \\ &\quad =- \biggl( \int_{a}^{mb}g(s)E_{\mu,\alpha,l}^{\gamma,\delta,k,c} \bigl(\omega s^{\mu};p\bigr)\,ds \biggr)^{\alpha}f(a) \\ & \qquad {}+\alpha \int _{a}^{mb} \biggl( \int_{t}^{mb}g(s)E_{\mu,\alpha,l}^{\gamma,\delta,k,c} \bigl(\omega s^{\mu};p\bigr)\,ds \biggr)^{\alpha-1}g(t)E_{\mu,\alpha,l}^{\gamma,\delta,k,c}\bigl(\omega t^{\mu };p \bigr)f(t)\,dt. \end{aligned}$$

Subtracting (9) from (8), we get (7) which is the required identity. □

If we take $m=1$ in (7), then we get the following identity for a convex function.

### Corollary 2.2

*Let* $f:[a,b]\subseteq[0,\infty)\rightarrow\mathbb{R}$ *be a differentiable function such that* $f'\in L_{1}[a,b]$ *with* $a< b$. *Also let* $g:[a,b]\rightarrow\mathbb{R}$ *be continuous on* $[a,b]$, *then the following identity for extended generalized fractional integral operators holds*:

10 $$\begin{aligned} & \biggl( \int_{a}^{b}g(s)E_{\mu,\alpha,l}^{\gamma,\delta,k,c} \bigl(\omega s^{\mu};p\bigr)\,ds \biggr)^{\alpha}\bigl[f(a)+f(b) \bigr] \\ &\qquad{} -\alpha \int _{a}^{b} \biggl( \int_{a}^{t}g(s)E_{\mu,\alpha,l}^{\gamma,\delta,k,c} \bigl(\omega s^{\mu};p\bigr)\,ds \biggr)^{\alpha-1} g(t)E_{\mu,\alpha,l}^{\gamma,\delta,k,c}\bigl(\omega t^{\mu };p\bigr)f(t)\,dt \\ &\qquad{} -\alpha \int_{a}^{b} \biggl( \int_{t}^{b}g(s)E_{\mu,\alpha,l}^{\gamma,\delta,k,c}\bigl( \omega s^{\mu};p\bigr)\,ds \biggr)^{\alpha -1}g(t)E_{\mu,\alpha,l}^{\gamma,\delta,k,c} \bigl(\omega t^{\mu };p\bigr)f(t)\,dt \\ &\quad = \int_{a}^{b} \biggl( \int_{a}^{t}g(s)E_{\mu,\alpha,l}^{\gamma,\delta,k,c} \bigl(\omega s^{\mu};p\bigr)\,ds \biggr)^{\alpha}f'(t)\,dt \\ &\qquad{} - \int_{a}^{b} \biggl( \int_{t}^{b}g(s)E_{\mu,\alpha,l}^{\gamma,\delta,k,c}\bigl( \omega s^{\mu};p\bigr)\,ds \biggr)^{\alpha}f'(t)\,dt. \end{aligned}$$

We use identity (7) to establish the following fractional integral inequality.

### Theorem 2.3

*Let* $f:[a,mb]\rightarrow\mathbb{R}$ *be a differentiable function such that* $f'\in L_{1}[a,mb]$ *with* $0\leq a< mb$. *Also let* $g:[a,mb]\rightarrow\mathbb{R}$ *be a continuous function on* $[a,mb]$. *If* $|f'|$ *is an* *m*-*convex function on* $[a,mb]$, *then the following inequality for extended generalized fractional integral operators holds*:

11 $$\begin{aligned} & \biggl\vert \biggl( \int_{a}^{mb}g(s)E_{\mu,\alpha,l}^{\gamma,\delta,k, c} \bigl(\omega s^{\mu}; p\bigr)\,ds \biggr)^{\alpha}\bigl(f(a)+f(mb) \bigr) \\ &\qquad{} -\alpha \int_{a}^{mb} \biggl( \int_{a}^{t}g(s)E_{\mu,\alpha,l}^{\gamma,\delta,k, c} \bigl(\omega s^{\mu}; p\bigr)\,ds \biggr)^{\alpha-1} g(t)E_{\mu,\alpha,l}^{\gamma,\delta,k, c}\bigl(\omega t^{\mu}; p\bigr)f(t)\,dt \\ &\qquad{} -\alpha \int_{a}^{mb} \biggl( \int_{t}^{mb}g(s)E_{\mu,\alpha,l}^{\gamma,\delta,k, c} \bigl(\omega s^{\mu}; p\bigr)\,ds \biggr)^{\alpha-1} g(t)E_{\mu,\alpha,l}^{\gamma,\delta,k, c}\bigl(\omega t^{\mu}; p\bigr)f(t)\,dt \biggr\vert \\ &\quad \leq\frac{(mb-a)^{\alpha+1} \Vert g \Vert _{\infty}^{\alpha}S^{\alpha }}{(\alpha+1)}\bigl( \bigl\vert f'(a) \bigr\vert +m \bigl\vert f'(b) \bigr\vert \bigr) \end{aligned}$$

*for* $k<\delta+\Re(\mu)$ *and* $\Vert g \Vert_{\infty}= \sup_{t\in[a,mb]}|g(t)|$.

### Proof

From Lemma 2.1, we have

12 $$\begin{aligned} & \biggl\vert \biggl( \int_{a}^{mb}g(s)E_{\mu,\alpha,l}^{\gamma,\delta,k, c} \bigl(\omega s^{\mu}; p\bigr)\,ds \biggr)^{\alpha}\bigl(f(a)+f(mb) \bigr) \\ &\qquad{} -\alpha \int_{a}^{mb} \biggl( \int_{a}^{t}g(s)E_{\mu,\alpha,l}^{\gamma,\delta,k, c} \bigl(\omega s^{\mu}; p\bigr)\,ds \biggr)^{\alpha-1} g(t)E_{\mu,\alpha,l}^{\gamma,\delta,k, c}\bigl(\omega t^{\mu}; p\bigr)f(t)\,dt \\ &\qquad{} -\alpha \int_{a}^{mb} \biggl( \int _{t}^{mb}g(s)E_{\mu,\alpha,l}^{\gamma,\delta,k, c} \bigl(\omega s^{\mu}; p\bigr)\,ds \biggr)^{\alpha-1} g(t)E_{\mu,\alpha,l}^{\gamma,\delta,k, c}\bigl(\omega t^{\mu}; p\bigr)f(t)\,dt \biggr\vert \\ & \quad \leq \int_{a}^{mb} \biggl\vert \int_{a}^{t}g(s)E_{\mu,\alpha,l}^{\gamma,\delta,k, c} \bigl(\omega s^{\mu}; p\bigr)\,ds \biggr\vert ^{\alpha } \bigl\vert f'(t) \bigr\vert \,dt \\ &\qquad{} + \int_{a}^{mb} \biggl\vert \int_{t}^{mb}g(s)E_{\mu,\alpha,l}^{\gamma,\delta,k, c} \bigl(\omega s^{\mu}; p\bigr)\,ds \biggr\vert ^{\alpha} \bigl\vert f'(t) \bigr\vert \,dt. \end{aligned}$$

Using absolute convergence of the Mittag-Leffler function and $\Vert g \Vert_{\infty}= \sup_{t\in[a,b]}|g(t)|$, we have

13 $$\begin{aligned} & \biggl\vert \biggl( \int_{a}^{mb}g(s)E_{\mu,\alpha,l}^{\gamma,\delta,k, c} \bigl(\omega s^{\mu}; p\bigr)\,ds \biggr)^{\alpha}\bigl(f(a)+f(mb) \bigr) \\ &\qquad{} -\alpha \int_{a}^{mb} \biggl( \int_{a}^{t}g(s)E_{\mu,\alpha,l}^{\gamma,\delta,k, c} \bigl(\omega s^{\mu}; p\bigr)\,ds \biggr)^{\alpha-1} g(t)E_{\mu,\alpha,l}^{\gamma,\delta,k, c}\bigl(\omega t^{\mu}; p\bigr)f(t)\,dt \\ &\qquad{} -\alpha \int_{a}^{mb} \biggl( \int_{t}^{mb}g(s)E_{\mu,\alpha,l}^{\gamma,\delta,k, c} \bigl(\omega s^{\mu}; p\bigr)\,ds \biggr)^{\alpha-1} g(t)E_{\mu,\alpha,l}^{\gamma,\delta,k, c}\bigl(\omega t^{\mu}; p\bigr)f(t)\,dt \biggr\vert \\ & \quad \leq \Vert g \Vert _{\infty}^{\alpha}S^{\alpha} \biggl( \int _{a}^{mb}(t-a)^{\alpha} \bigl\vert f'(t) \bigr\vert \,dt+ \int_{a}^{mb}(mb-t)^{\alpha } \bigl\vert f'(t) \bigr\vert \,dt \biggr). \end{aligned}$$

Since $|f'|$ is an *m*-convex function, we have

14 $$ \bigl\vert f'(t) \bigr\vert \leq \frac{mb-t}{mb-a} \bigl\vert f'(a) \bigr\vert +m \frac{t-a}{mb-a} \bigl\vert f'(b) \bigr\vert $$

for $t\in[a,mb]$.

Using (14) in (13), we have

15 $$\begin{aligned} & \biggl\vert \biggl( \int_{a}^{mb}g(s)E_{\mu,\alpha,l}^{\gamma,\delta,k, c} \bigl(\omega s^{\mu}; p\bigr)\,ds \biggr)^{\alpha}\bigl(f(a)+f(mb) \bigr) \\ &\qquad{} -\alpha \int_{a}^{mb} \biggl( \int_{a}^{t}g(s)E_{\mu,\alpha,l}^{\gamma,\delta,k, c} \bigl(\omega s^{\mu}; p\bigr)\,ds \biggr)^{\alpha-1} g(t)E_{\mu,\alpha,l}^{\gamma,\delta,k, c}\bigl(\omega t^{\mu}; p\bigr)f(t)\,dt \\ &\qquad{} -\alpha \int_{a}^{mb} \biggl( \int_{t}^{mb}g(s)E_{\mu,\alpha,l}^{\gamma,\delta,k, c} \bigl(\omega s^{\mu}; p\bigr)\,ds \biggr)^{\alpha-1} g(t)E_{\mu,\alpha,l}^{\gamma,\delta,k, c}\bigl(\omega t^{\mu}; p\bigr)f(t)\,dt \biggr\vert \\ & \quad \leq \Vert g \Vert _{\infty}^{\alpha}S^{\alpha} \biggl( \int _{a}^{mb}(t-a)^{\alpha} \biggl( \frac{mb-t}{mb-a} \bigl\vert f'(a) \bigr\vert +m \frac {t-a}{mb-a} \bigl\vert f'(b) \bigr\vert \biggr)\,dt \\ &\qquad{} + \int_{a}^{mb}(mb-t)^{\alpha} \biggl( \frac {mb-t}{mb-a} \bigl\vert f'(a) \bigr\vert +m \frac{t-a}{mb-a} \bigl\vert f'(b) \bigr\vert \biggr)\,dt \biggr) . \end{aligned}$$

After simple calculation of the above inequality, we get (11) which is required. □

If we take $m=1$ in (11), then we get the following result for a convex function.

### Corollary 2.4

*Let* $f:[a,b]\subseteq[0,\infty)\rightarrow\mathbb{R}$ *be a differentiable function such that* $f'\in L_{1}[a,b]$ *with* $a< b$. *Also let* $g:[a,b]\rightarrow\mathbb{R}$ *be a continuous function on* $[a,b]$. *If* $|f'|$ *is a convex function on* $[a,b]$, *then the following inequality for extended generalized fractional integral operators holds*:

16 $$\begin{aligned} & \biggl\vert \biggl( \int_{a}^{b}g(s)E_{\mu,\alpha,l}^{\gamma,\delta,k,c} \bigl(\omega s^{\mu};p\bigr)\,ds \biggr)^{\alpha}\bigl[f(a)+f(b) \bigr] \\ &\quad{} -\alpha \int_{a}^{b} \biggl( \int_{a}^{t}g(s)E_{\mu,\alpha,l}^{\gamma,\delta,k,c} \bigl(\omega s^{\mu};p\bigr)\,ds \biggr)^{\alpha-1} g(t)E_{\mu,\alpha,l}^{\gamma,\delta,k,c}\bigl(\omega t^{\mu };p\bigr)f(t)\,dt \\ &\quad{} -\alpha \int_{a}^{b} \biggl( \int_{t}^{b}g(s)E_{\mu,\alpha,l}^{\gamma,\delta,k,c}\bigl( \omega s^{\mu};p\bigr)\,ds \biggr)^{\alpha -1}g(t)E_{\mu,\alpha,l}^{\gamma,\delta,k,c} \bigl(\omega t^{\mu };p\bigr)f(t)\,dt \biggr\vert \\ & \leq\frac{(b-a)^{\alpha+1} \Vert g \Vert _{\infty}^{\alpha}S^{\alpha }}{(\alpha+1)}\bigl[ \bigl\vert f'(a) \bigr\vert + \bigl\vert f'(b) \bigr\vert \bigr] \end{aligned}$$

*for* $k<\delta+\Re(\mu)$ *and* $\Vert g \Vert_{\infty}= \sup_{t\in[a,b]}|g(t)|$.

### Remark 2.5

In Theorem 2.3.

(i) If we put $p=0$, then we get [6, Theorem 3.2].
(ii) If we put $\omega=p=0$ and $m=1$, then we get [13, Theorem 6].
(iii) If we take $\omega=p=0$, $m=1$, $\alpha=\frac{\mu }{k}$, and $g(s)=1$, then we get [8, Corollary 2.3].
(iv) For $g(s)=1$ along with $\omega=p=0$, $m=1$, and $\alpha =\mu$, we get [13, Corollary 2].

### Remark 2.6

In Corollary 2.4.

(i) If we put $p=0$, then we get [1, Theorem 3.2].
(ii) If we put $\omega=p=0$, then we get [13, Theorem 6].
(iii) For $\omega=p=0$, $\alpha=\frac{\mu}{k}$, and $g(s)=1$, we get [8, Corollary 2.3].
(iv) For $g(s)=1$ along with $\omega=p=0$, we get [13, Corollary 2].

Next we give the following fractional integral inequality.

### Theorem 2.7

*Let* $f:[a,mb]\rightarrow\mathbb{R}$ *be a differentiable function such that* $f\in L_{1}[a,mb]$ *with* $0\leq a< mb$. *Also let* $g:[a,mb]\rightarrow\mathbb{R}$ *be a continuous function on* $[a,mb]$. *If* $|f'|^{q}$ *is a convex function on* $[a,mb]$, *then for* $q>0$ *the following inequality for extended generalized fractional integral operators holds*:

17 $$\begin{aligned} & \biggl\vert \biggl( \int_{a}^{mb}g(s)E_{\mu,\alpha,l}^{\gamma,\delta,k, c} \bigl(\omega s^{\mu}; p\bigr)\,ds \biggr)^{\alpha}\bigl(f(a)+f(mb) \bigr) \\ &\qquad{} -\alpha \int_{a}^{mb} \biggl( \int_{a}^{t}g(s)E_{\mu,\alpha,l}^{\gamma,\delta,k, c} \bigl(\omega s^{\mu}; p\bigr)\,ds \biggr)^{\alpha-1} g(t)E_{\mu,\alpha,l}^{\gamma,\delta,k, c}\bigl(\omega t^{\mu}; p\bigr)f(t)\,dt \\ &\qquad{} -\alpha \int_{a}^{mb} \biggl( \int_{t}^{mb}g(s)E_{\mu,\alpha,l}^{\gamma,\delta,k, c} \bigl(\omega s^{\mu}; p\bigr)\,ds \biggr)^{\alpha-1} g(t)E_{\mu,\alpha,l}^{\gamma,\delta,k, c}\bigl(\omega t^{\mu}; p\bigr)f(t)\,dt \biggr\vert \\ &\quad \leq\frac{2(mb-a)^{\alpha+1} \Vert g \Vert _{\infty}^{\alpha}S^{\alpha }}{(\alpha p+1)^{\frac{1}{q}}} \biggl(\frac { \vert f'(a) \vert ^{q}+m \vert f'(b) \vert ^{q}}{2} \biggr)^{\frac{1}{q}} \end{aligned}$$

*for* $k<\delta+\Re(\mu)$ *and* $\Vert g \Vert_{\infty}= \sup_{t\in[a,mb]}|g(t)|$ *and* $\frac{1}{p}+\frac{1}{q}=1$.

### Proof

From Lemma 2.1 and by using Hölder’s inequality, we have

18 $$\begin{aligned} & \biggl\vert \biggl( \int_{a}^{mb}g(s)E_{\mu,\alpha,l}^{\gamma,\delta,k, c} \bigl(\omega s^{\mu}; p\bigr)\,ds \biggr)^{\alpha}\bigl(f(a)+f(mb) \bigr) \\ &\qquad{} -\alpha \int_{a}^{mb} \biggl( \int_{a}^{t}g(s)E_{\mu,\alpha,l}^{\gamma,\delta,k, c} \bigl(\omega s^{\mu}; p\bigr)\,ds \biggr)^{\alpha-1} g(t)E_{\mu,\alpha,l}^{\gamma,\delta,k, c}\bigl(\omega t^{\mu}; p\bigr)f(t)\,dt \\ &\qquad{} -\alpha \int_{a}^{mb} \biggl( \int_{t}^{mb}g(s)E_{\mu,\alpha,l}^{\gamma,\delta,k, c} \bigl(\omega s^{\mu}; p\bigr)\,ds \biggr)^{\alpha-1} g(t)E_{\mu,\alpha,l}^{\gamma,\delta,k, c}\bigl(\omega t^{\mu}; p\bigr)f(t)\,dt \biggr\vert \\ & \quad \leq \biggl( \int_{a}^{mb} \biggl\vert \int_{a}^{t}g(s)E_{\mu,\alpha,l}^{\gamma,\delta,k, c} \bigl(\omega s^{\mu}; p\bigr)\,ds \biggr\vert ^{\alpha p}\,dt \biggr)^{\frac{1}{p}} \biggl( \int_{a}^{mb} \bigl\vert f'(t) \bigr\vert ^{q}\,dt \biggr)^{\frac{1}{q}} \\ &\qquad{} + \biggl( \int_{a}^{mb} \biggl\vert \int_{t}^{mb}g(s)E_{\mu,\alpha,l}^{\gamma,\delta,k, c} \bigl(\omega s^{\mu}; p\bigr)\,ds \biggr\vert ^{\alpha p}\,dt \biggr)^{\frac{1}{p}} \biggl( \int_{a}^{mb} \bigl\vert f'(t) \bigr\vert ^{q}\,dt \biggr)^{\frac{1}{q}}. \end{aligned}$$

Using absolute convergence of the Mittag-Leffler function and $\Vert g \Vert_{\infty}= \sup_{t\in[a,b]}|g(t)|$, we have

19 $$\begin{aligned} & \biggl\vert \biggl( \int_{a}^{mb}g(s)E_{\mu,\alpha,l}^{\gamma,\delta,k, c} \bigl(\omega s^{\mu}; p\bigr)\,ds \biggr)^{\alpha}\bigl(f(a)+f(mb) \bigr) \\ &\qquad{} -\alpha \int_{a}^{mb} \biggl( \int_{a}^{t}g(s)E_{\mu,\alpha,l}^{\gamma,\delta,k, c} \bigl(\omega s^{\mu}; p\bigr)\,ds \biggr)^{\alpha-1} g(t)E_{\mu,\alpha,l}^{\gamma,\delta,k, c}\bigl(\omega t^{\mu}; p\bigr)f(t)\,dt \\ &\qquad{} -\alpha \int_{a}^{mb} \biggl( \int _{t}^{mb}g(s)E_{\mu,\alpha,l}^{\gamma,\delta,k, c} \bigl(\omega s^{\mu}; p\bigr)\,ds \biggr)^{\alpha-1} g(t)E_{\mu,\alpha,l}^{\gamma,\delta,k, c}\bigl(\omega t^{\mu}; p\bigr)f(t)\,dt \biggr\vert \\ & \quad \leq \Vert g \Vert _{\infty}^{\alpha}S^{\alpha} \biggl( \biggl( \int _{a}^{mb} \vert t-a \vert ^{\alpha p}\,dt \biggr)^{\frac{1}{p}} \\ &\qquad {}+ \biggl( \int _{a}^{mb} \vert mb-t \vert ^{\alpha p}\,dt \biggr)^{\frac{1}{p}} \biggr) \biggl( \int _{a}^{mb} \bigl\vert f'(t) \bigr\vert ^{q}\,dt \biggr)^{\frac{1}{q}}. \end{aligned}$$

Since $|f'(t)|^{q}$ is an *m*-convex function, we have

20 $$ \bigl\vert f'(t) \bigr\vert ^{q}\leq \frac{mb-t}{mb-a} \bigl\vert f'(a) \bigr\vert ^{q}+m \frac{t-a}{mb-a} \bigl\vert f'(b) \bigr\vert ^{q}. $$

Using (20) in (19), we have

21 $$\begin{aligned} & \biggl\vert \biggl( \int_{a}^{mb}g(s)E_{\mu,\alpha,l}^{\gamma,\delta,k, c} \bigl(\omega s^{\mu}; p\bigr)\,ds \biggr)^{\alpha}\bigl(f(a)+f(mb) \bigr) \\ &\qquad{} -\alpha \int_{a}^{mb} \biggl( \int_{a}^{t}g(s)E_{\mu,\alpha,l}^{\gamma,\delta,k, c} \bigl(\omega s^{\mu}; p\bigr)\,ds \biggr)^{\alpha-1} g(t)E_{\mu,\alpha,l}^{\gamma,\delta,k, c}\bigl(\omega t^{\mu}; p\bigr)f(t)\,dt \\ &\qquad{} -\alpha \int_{a}^{mb} \biggl( \int_{t}^{mb}g(s)E_{\mu,\alpha,l}^{\gamma,\delta,k, c} \bigl(\omega s^{\mu}; p\bigr)\,ds \biggr)^{\alpha-1} g(t)E_{\mu,\alpha,l}^{\gamma,\delta,k, c}\bigl(\omega t^{\mu}; p\bigr)f(t)\,dt \biggr\vert \\ & \quad \leq \Vert g \Vert _{\infty}^{\alpha}S^{\alpha} \biggl( \biggl( \int _{a}^{mb} \vert t-a \vert ^{\alpha p}\,dt \biggr)^{\frac{1}{p}}+ \biggl( \int _{a}^{mb} \vert mb-t \vert ^{\alpha p}\,dt \biggr)^{\frac{1}{p}} \biggr) \\ & \qquad {}\times \biggl( \int_{a}^{mb}\frac{mb-t}{mb-a} \bigl\vert f'(a) \bigr\vert ^{q}+m\frac {t-a}{mb-a} \bigl\vert f'(b) \bigr\vert ^{q} \biggr)^{\frac{1}{q}} . \end{aligned}$$

After simple calculation of the above inequality, we get (17) which is required. □

If we take $m=1$ in (17), then we get the following result for a convex function.

### Corollary 2.8

*Let* $f:[a,b]\subseteq[0,\infty)\rightarrow\mathbb{R}$ *be a differentiable function such that* $f'\in L_{1}[a,b]$ *with* $a< b$. *Also let* $g:[a,b]\rightarrow\mathbb{R}$ *be a continuous function on* $[a,b]$. *If* $|f'|^{q}$ *is a convex function on* $[a,b]$, *then for* $q>0$ *the following inequality for extended generalized fractional integral operators holds*:

22 $$\begin{aligned} & \biggl\vert \biggl( \int_{a}^{b}g(s)E_{\mu,\alpha,l}^{\gamma,\delta,k,c} \bigl(\omega s^{\mu};p\bigr)\,ds \biggr)^{\alpha}\bigl[f(a)+f(b) \bigr] \\ &\qquad{} -\alpha \int_{a}^{b} \biggl( \int_{a}^{t}g(s)E_{\mu,\alpha,l}^{\gamma,\delta,k,c} \bigl(\omega s^{\mu};p\bigr)\,ds \biggr)^{\alpha -1}g(t)E_{\mu,\alpha,l}^{\gamma,\delta,k,c} \bigl(\omega t^{\mu};p\bigr)f(t)\,dt \\ &\qquad{} -\alpha \int_{a}^{b} \biggl( \int_{t}^{b}g(s)E_{\mu,\alpha,l}^{\gamma,\delta,k,c}\bigl( \omega s^{\mu};p\bigr)\,ds \biggr)^{\alpha -1}g(t)E_{\mu,\alpha,l}^{\gamma,\delta,k,c} \bigl(\omega t^{\mu };p\bigr)f(t)\,dt \biggr\vert \\ &\quad \leq\frac{2(b-a)^{\alpha+1} \Vert g \Vert _{\infty}^{\alpha}S^{\alpha }}{(\alpha p+1)^{\frac{1}{q}}} \biggl[\frac { \vert f'(a) \vert ^{q}+ \vert f'(b) \vert ^{q}}{2} \biggr]^{\frac{1}{q}} \end{aligned}$$

*for* $k<\delta+\Re(\mu)$ *and* $\Vert g \Vert_{\infty}= \sup_{t\in[a,b]}|g(t)|$ *and* $\frac{1}{p}+\frac{1}{q}=1$.

### Remark 2.9

In Theorem 2.7.

(i) If we put $p=0$, then we get [6, Theorem 3.6].
(ii) If we put $\omega=p=0$ and $m=1$, then we get [13, Theorem 7].
(iii) If we take $\omega=p=0$, $m=1$ along with $\alpha=\frac {\mu}{k}$, then we get [8, Theorem 2.5].
(iv) If we take $g(s)=1$, $m=1$, and $\omega=p=0$, then we get [5, Theorem 2.3].
(v) If we put $\omega=p=0$, $m=1$, and $\alpha=1$, then we get [5, Corollary 3].

### Remark 2.10

In Corollary 2.8.

(i) If we put $p=0$, then we get [1, Theorem 3.5].
(ii) If we put $\omega=p=0$, then we get [13, Theorem 7].
(iii) If we put $\omega=p=0$, $\alpha=1$, then we get [13, Corollary 3].
(iv) If we take $\omega=p=0$ along with $\alpha=\frac{\mu }{k}$, then we get [8, Theorem 2.5].
(v) If we take $g(s)=1$ and $\omega=p=0$, then we get [5, Theorem 2.3].

In the next result we give Hadamard type inequalities for *m*-convex functions via an extended Mittag-Leffler function.

### Theorem 2.11

*Let* $f:[a,mb]\rightarrow\mathbb{R}$ *be a function such that* $f\in L_{1}[a,mb]$ *with* $0\leq a< mb$. *If* *f* *is* *m*-*convex on* $[a,mb]$, *then the following inequalities for extended generalized fractional integral operators hold*:

23 $$\begin{aligned} &2f \biggl(\frac{a+mb}{2} \biggr)C_{\alpha, (\frac {a+mb}{2} )^{+}}(mb;p) \\ &\quad \leq \bigl( \epsilon_{\mu,\alpha,l,\omega',(\frac {a+mb}{2})^{+}}^{\gamma,\delta,k, c}f \bigr) (mb;p) + m^{\alpha+1} \bigl( \epsilon_{\mu,\alpha,l,m^{\mu}\omega',(\frac {a+mb}{2m})^{-}}^{\gamma,\delta,k, c}f \bigr) \biggl( \frac {a}{m};p \biggr) \\ &\quad \leq\frac{1}{mb-a} \biggl( f(a)-m^{2}f \biggl(\frac{a}{m^{2}} \biggr) \biggr)C_{\alpha+1, (\frac{a+mb}{2} )^{+}}(mb;p) \\ &\qquad{} +m^{\alpha+1} \biggl( f(b)+mf \biggl(\frac{a}{m^{2}} \biggr) \biggr)C_{\alpha, (\frac{a+mb}{2m} )^{-}} \biggl(\frac {a}{m};p \biggr), \end{aligned}$$

*where* $\omega'=\frac{2^{\mu}\omega}{(mb-a)^{\mu}}$.

### Proof

Since *f* is an *m*-convex function, we have

24 $$ 2f \biggl(\frac{a+mb}{2} \biggr)\leq f \biggl( \frac{t}{2}a+\frac {2-t}{2}mb \biggr)+mf \biggl(\frac{2-t}{2m}a+ \frac{t}{2}b \biggr). $$

Also from *m*-convexity of *f*, we have

25 $$\begin{aligned} &f \biggl(\frac{t}{2}a+m\frac{2-t}{2}b \biggr)+mf \biggl( \frac {2-t}{2m}a+\frac{t}{2}b \biggr) \\ &\quad \leq\frac{t}{2} \biggl(f(a)-m^{2}f \biggl(\frac{a}{m^{2}} \biggr) \biggr)+m \biggl(f(b)+mf \biggl(\frac{a}{m^{2}} \biggr) \biggr). \end{aligned}$$

Multiplying (24) by $t^{\alpha-1}E_{\mu,\alpha,l}^{\gamma,\delta,k, c}(\omega t^{\mu}; p)$ on both sides and then integrating over $[0,1]$, we have

26 $$\begin{aligned} &2f \biggl(\frac{a+mb}{2} \biggr) \int_{0}^{1}t^{\alpha-1}E_{\mu,\alpha,l}^{\gamma,\delta,k, c} \bigl(\omega t^{\mu}; p\bigr)\,dt \\ &\quad \leq \int_{0}^{1}t^{\alpha-1}E_{\mu,\alpha,l}^{\gamma,\delta,k, c} \bigl(\omega t^{\mu}; p\bigr)f \biggl(\frac{t}{2}a+ \frac{2-t}{2}mb \biggr)\,dt \\ &\qquad{} +m \int_{0}^{1}t^{\alpha-1}E_{\mu,\alpha,l}^{\gamma,\delta,k, c} \bigl(\omega t^{\mu}; p\bigr)f \biggl(\frac{2-t}{2m}a+ \frac{t}{2}b \biggr)\,dt. \end{aligned}$$

Putting $u=\frac{t}{2}a+\frac{2-t}{2}mb$ and $v=\frac {2-t}{2m}a+\frac{t}{2}b$ in (26), we have

$$\begin{aligned} &2f \biggl(\frac{a+mb}{2} \biggr) \int_{\frac {a+mb}{2}}^{mb}(mb-u)^{\alpha-1} E_{\mu,\alpha,l}^{\gamma,\delta,k, c}\bigl(\omega' (mb-u)^{\mu}; p\bigr)\,du \\ & \quad \leq \int_{\frac{a+mb}{2}}^{mb}(mb-u)^{\alpha-1}E_{\mu,\alpha,l}^{\gamma,\delta,k, c} \bigl(\omega' (mb-u)^{\mu}; p\bigr)f(u)\,du \\ &\qquad{} +m^{\alpha+1} \int_{\frac{a}{m}}^{\frac{a+mb}{2m}} \biggl(v-\frac {a}{m} \biggr)^{\alpha-1}E_{\mu,\alpha,l}^{\gamma,\delta,k, c} \biggl(m^{\mu} \omega'\biggl(v-\frac{a}{m}\biggr)^{\mu}:p \biggr)f(v)\,dv . \end{aligned}$$

By using (3), (4), and (5) we get the first inequality of (23).

Now multiplying (25) by $t^{\alpha-1}E_{\mu,\alpha,l}^{\gamma,\delta,k, c}(\omega t^{\mu}; p)$ on both sides and then integrating over $[0,1]$, we have

27 $$\begin{aligned} & \int_{0}^{1}t^{\alpha-1}E_{\mu,\alpha,l}^{\gamma,\delta,k, c} \bigl(\omega t^{\mu}; p\bigr)f \biggl(\frac{t}{2}a+m \frac{2-t}{2}b \biggr)\,dt \\ &\qquad{} +m \int_{0}^{1}t^{\alpha-1}E_{\mu,\alpha,l}^{\gamma,\delta,k, c} \bigl(\omega t^{\mu}; p\bigr)f \biggl(\frac{2-t}{2m}a+ \frac{t}{2}b \biggr) \\ & \quad \leq\frac{1}{2} \biggl(f(a)-m^{2}f\biggl(\frac{a}{m^{2}} \biggr) \biggr) \int _{0}^{1}t^{\alpha}E_{\mu,\alpha,l}^{\gamma,\delta,k, c} \bigl(\omega t^{\mu}; p\bigr)\,dt \\ &\qquad{} +m \biggl(f(b)+mf\biggl(\frac{a}{m^{2}}\biggr) \biggr) \int_{0}^{1}t^{\alpha -1}E_{\mu,\alpha,l}^{\gamma,\delta,k, c} \bigl(\omega t^{\mu}; p\bigr)\,dt. \end{aligned}$$

Putting $u=\frac{t}{2}a+m\frac{2-t}{2}b$ and $v=\frac {2-t}{2m}a+\frac{t}{2}b$ in (27), we have

28 $$\begin{aligned} & \int_{\frac{a+mb}{2}}^{mb}(mb-u)^{\alpha-1}E_{\mu,\alpha,l}^{\gamma,\delta,k, c} \bigl(\omega' (mb-u)^{\mu};p\bigr)f(u)\,du \\ &\qquad{} + \int_{\frac{a}{m}}^{\frac{a+mb}{2m}} \biggl(v-\frac{a}{m} \biggr)^{\alpha-1}E_{\mu,\alpha,l}^{\gamma,\delta,k, c}\biggl(m^{\mu} \omega' \biggl(v-\frac{a}{m} \biggr)^{\mu};p \biggr)f(v)\,dv \\ &\quad \leq\frac{1}{2} \biggl( f(a)-m^{2}f\biggl(\frac{a}{m^{2}} \biggr) \biggr) \int _{\frac{a+mb}{2}}^{mb}(mb-u)^{\alpha}E_{\mu,\alpha,l}^{\gamma,\delta,k, c} \bigl(\omega' (mb-u)^{\mu};p \bigr)\,dt \\ &\qquad{} +m^{\alpha+1} \biggl( f(b)+mf\biggl(\frac{a}{m^{2}}\biggr) \biggr) \\ &\qquad {}\times \int_{\frac {a}{m}}^{\frac{a+mb}{2m}} \biggl(v-\frac{a}{m} \biggr)^{\alpha -1}E_{\mu,\alpha,l}^{\gamma,\delta,k, c}\biggl(m^{\mu} \omega' \biggl(v-\frac{a}{m} \biggr)^{\mu};p \biggr)\,dt. \end{aligned}$$

By using (3), (4), and (6), we get the second inequality of (23). □

If we take $m=1$ in (23), then we get the following Hadamard type inequality for a convex function.

### Corollary 2.12

*Let* $f:[a,b]\subseteq[0,\infty)\rightarrow\mathbb{R}$ *be a function such that* $f\in L_{1}[a,b]$ *with* $a< b$. *If* *f* *is convex on* $[a,b]$, *then the following inequalities for extended generalized fractional integral operators hold*:

29 $$\begin{aligned} &f \biggl(\frac{a+b}{2} \biggr)C_{\alpha, (\frac{a+b}{2} )^{+}}(b;p) \\ &\quad \leq \bigl[ \bigl( \epsilon_{\mu,\alpha,l,\omega ',(\frac{a+b}{2})^{+}}^{\gamma,\delta,k,c}f \bigr) (b;p) + \bigl( \epsilon_{\mu,\alpha,l,\omega',(\frac {a+b}{2})_{-}}^{\gamma,\delta,k,c}f \bigr) (a;p) \bigr] \\ &\quad \leq\frac{f(a)+f(b)}{2}C_{\alpha, (\frac{a+b}{2} )^{-}}(a;p), \end{aligned}$$

*where* $\omega'=\frac{2^{\mu}\omega}{(b-a)^{\mu}}$.

### Remark 2.13

In Theorem 2.11.

(i) If we put $p=0$, then we get [6, Theorem 3.10].
(ii) If we put $\omega=p=0$, $m=1$, and $\alpha=1$, then we get the classical Hadamard inequality.

### Remark 2.14

In Corollary 2.12.

(i) If we put $p=0$, then we get [1, Theorem 3.9].
(ii) If we put $\omega=p=0$ and $\alpha=1$, then we get the classical Hadamard inequality.
(iii) If we take $\omega=p=0$, then we get [14, Theorem 4].

## Concluding remarks

We have investigated more general fractional integral inequalities. By selecting specific values of parameters quite interesting results can be obtained. For example selecting $p=0$, fractional integral inequalities for fractional integral operators defined by Salim and Faraj in [12], selecting $l=\delta=1$, fractional integral inequalities for fractional integral operators defined by Rahman et al. in [11], selecting $p=0$ and $l=\delta=1$, fractional integral inequalities for fractional integral operators defined by Shukla and Prajapati in [15] (see also [16]), selecting $p=0$ and $l=\delta=k=1$, fractional integral inequalities for fractional integral operators defined by Prabhakar in [10], selecting $p=\omega=0$, fractional integral inequalities for Riemann–Liouville fractional integral operators.
